# 

**Published:** 1885-07

**Authors:** 


### CONTENTS.

ORIGINAL ARTICLES: *Pages.*
Surgical Fracture of Femur. A Case, by R. M. Swearingen, M. D., Austin, Texas ........... 1
Premature Symptomatic Alopecia. By J. A. Abney, M. D., Lufkin, Texas ............... ... 3
Incision of the Sphincter Vaginæ, in Certain Cases. By W. A. Morris, M. D., Austin, Texas...... 4
Some Therapeutical Indications for the Use of Jaborandi. By Q. C. Smith, M. D., Austin, Tex. 9
Case of Crypsorchis. By C. S. Gwyn, M. D., Galveston, Texas .............................. 12
Typhoid Fever. A Case with Unusual Complications. By T. J. Bennett, M. D., Austin, Tex. 13
CULLINGS FROM CONTEMPORARIES:
Rupture of the Perineum. From a paper by Frank Hastings Hamilton, M. D.................... 15
Intra-peritoneal Adhesions. From a paper by B. E. Hadra, M. D. .......................... 18
Cure of Aneurism by Insertion of Wire ......... 21
Lupus Cured by Arsenic Internally .............. 21
A Case of Double Uterus with a Fœtus in each. A paper by W. E. Lane, M. D. ................ 22
All Wrong. Extracts from a paper on Clothing, by Dr. Thos. Herbert, New Iberia, La. ..... . 24
The Curability of Consumption—Remarks on Prof. Jacoud's New Book .......................... 26
The Latest Surgical Triumph — the Transplantation of Eye from Rabbit to Man.............. 27
CORRESPONDENCE:
Our Chicago Letter. By Dr. J. W. McLaughlin .. 28
EDITORIAL:
Launched ........................................ 30
Heresy, False Doctrine and Schism.............. 33
Rationale of the Action of Cocaine............... 37
Salve for a Sore Head .......................... 40
Hold up Our Hands ............ ............ 42
A Meaningless Act, and What Came of It ........ 42
An Important Texas Work ........................ 47
The New Louisiana Quarantine................... 48
SOCIETY NOTES:
The Ellis County Medical Society; The Austin Club; Transactions Texas State Medical Association; The Comanche Co. Medical Society 52
MELANGE:
Christened; Precautions Against Cholera; Todgers Redivivus ............................. 53
ADVERTISERS' NOTICES:
College Physicians and Surgeons; The Old Reliable; Tulane University; University of Louisville; University of Nashville; Battle & Co. 53

